# Numerical modelling on geotechnical features of soil mixture using recycled tire crumb to strengthen the seismic isolation in building

**DOI:** 10.1038/s41598-023-50741-w

**Published:** 2024-01-02

**Authors:** J. Cici Jennifer Raj, M. Vinod Kumar, Mehmet Serkan Kırgız, N. Nagaprasad, Krishnaraj Ramaswamy

**Affiliations:** 1 Department of Civil Engineering, Vel Tech Rangarajan Dr. Sagunthala R&D Institute of Science and Technology, Chennai, India; 2 Northwestern University, Chicago, IL 60208-001 USA; 3 Department of Mechanical Engineering, ULTRA College of Engineering and Technology, Madurai, Tamilnadu 625104 India; 4 Centre for Excellence-Indigenous Knowledge, Innovative Technology Transfer and Entrepreneurship, Dambi Dollo University, Dambi Dollo, Ethiopia; 5 Department of Mechanical Engineering, College of Engineering and Technology, Dambi Dollo University, Dambi Dollo, Ethiopia

**Keywords:** Energy science and technology, Engineering, Materials science, Physics

## Abstract

In this research, the performance pertaining to tire crumb obtained from scrap tire processing plants is discussed. These tire crumbs are blended with soil at a 30% ratio. When subjected to seismic load, the performance of the 30% tire crumb combination is superior to the 0% tire crumb combination. The investigation is classified into two phases. Phase 1 of the study involves conducting an experimental investigation by applying cyclic loads to a model footing that was resting on the soil with and without tire crumbs. This study reveals that a 30% tire crumb combination achieves optimum energy absorption and minimal footing stiffness, which is a crucial component needed for base isolation. Additionally, using the PLAXIS 2D software package, finite element analysis was carried out during the second phase of the study. For this study, a three-story residential building close to the border between India and Nepal is used. Three different disastrous seismic excitations are applied to the building. From this analytical analysis, it is reported that a 60–70% reduction in acceleration is attained for 30% tire crumb combination with soil. Therefore, from the two phases, it is evaluated that the inclusion of tire crumbs with soil is an excellent seismic base isolation material.

## Introduction

The concept of seismic isolation is that the structure/building is protected by isolating the substructure from the superstructure; thereby, the upcoming seismic waves caused due to earthquakes are absorbed^1–4^. The aspect of reducing seismic demand than capacity is applicable in this technique of seismic isolation irrespective of other earthquake-resistant techniques^5,6^. In spite of the need for seismic isolation, this is widely adaptable in developed countries and least applied in developing countries due to various^7–10^. Literally, the technology of seismic isolation is quite costlier and even developed countries like Japan have started to utilize recyclable and natural materials/industrial by-products for seismic isolation. Many research is being conducted to identify a suitable recyclable material for base isolation^11–15^. Additionally, there is a needing of understanding for solving the seismic isolation problem widely, because the problem is also related to the soil-structure interaction (SSI). In that broad field, there could also find plenty of much research which was highlighted the problem and suggested a number of ways for solving the problem^16^. For instance, Firoj et al. studied effect of nonlinear soil–structure interaction and lateral stiffness on seismic performance of mid-rise RC building. They have found that lateral stiffness and SSI significantly affect the seismic performance in mid-rise buildings^17^. Bhuguna and Firoj reported another current work entitled “numerical simulation of seismic response of slope–foundation–structure interaction for mid-rise RC buildings at various locations. They unveiled that the building was built on the slope is much more vulnerable to rocking failure than that of the building resided at the top and bottom of the slope. Considered the increase in slope angle, the rocking of the foundation is also increasing. Tasleem et al. presented their results about effect of open-ground storey (OGS) on RC frame buildings incorporating soil–structure interaction. The most outstanding result concluded by them is that the difference in the period of the OGS construction with no strut member is higher than the OGS construction with strut member. They stated that strut member is important for the OGS construction^18^. In last study, Bhuguna and Firoj emphasized soil–structure interaction for nuclear structure in terms of nonlinear seismic performance. They inferred that the concrete damage plasticity (CDP) model showed the requirement to reconsider the nonlinearity of nuclear structure along with the nonlinearity of soil^19^. The centrifuge modelling with an earthquake shaker under a 50 g acceleration field utilized was studied to replicate the actual nonlinear dynamic response characteristics of RSM and subsurface in a coupled soil foundation-structure system. From the investigation, it is observed that 40–50% reduction in seismic demand is attained^20,21^.

**Figure 1:**
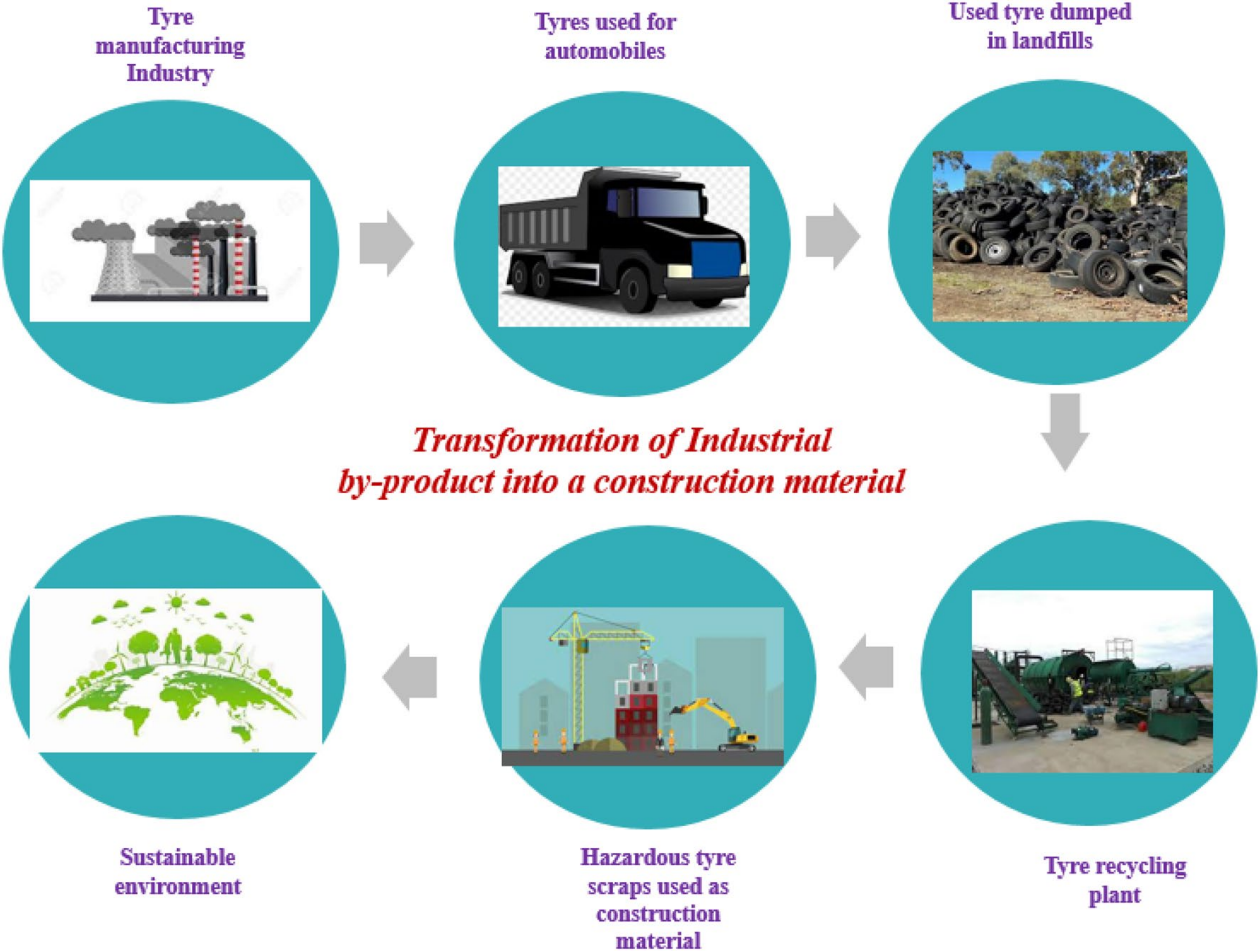
Scope of the research.

Nevertheless, approximately 16 countries are utilizing waste materials for recycling exclusively for civil engineering applications. Scrap tires are the exclusive waste material required to be used for construction as the dumping of tire causes huge distress to the environment and society^22^. Tire in the rubber and the steel reinforcing cords inbuilt it together behaves like a conventional rubber bearing^23,24^. The scrap tire could be used as bearings or tirecrumb^19^. The scope of the research is to ‘transform an industrial by-product into a construction material’, as shown in Fig. 1.

In this present research, tire crumbs, a waste hazardous material (by-product), are utilized, and its performance under dynamic loads is studied. For instance, the tires manufactured from tire manufacturing industries are used in automobiles such as trucks, buses and cars^25–27^. Non-Governmental Organizations report that around 25 million waste tires are dumped in landfills and counsel to implement these tires as a recyclable material exclusively for construction^28–38^. From the investigation, Researchers found that the higher reduction caused by shear failure is disregarded while looking at footing failure in the analysis^39–41^.

The damping property of the tire-soil mix was studied by the researchers using a triaxial-compression test. From the study, the authors concluded that a significant reduction in shear strength was achieved. Furthermore, the authors reported that an increase in tire content in soils increases the damping factor predominantly^42–45^. The geotechnical properties, such as compressibility and compaction, were studied for a soil–tire mix. The researchers concluded that the combination of a tire of 30% with soil has prominent results, and subsequently, satisfactory shear strength is attained^46–48^. The performance of the soil–tire mix with concrete was studied. The authors reported that significant attainment of durability was achieved^49–52^. The properties of rubber soil mixtures have been studied to determine the energy absorption capacity of medium-rise buildings against seismic forces. The authors conducted an undrained triaxial test and a direct shear test to identify the best and most appropriate size of tire crumbs.

Furthermore, a numerical analysis was performed, and the authors reported that the acceleration and inter-storey drift at the floor level could be reduced by 40–50% with the use of RSM^53^. The preliminary investigation was conducted to determine the properties of Ottawa sand, and tire chips in the proportions of 70% Ottawa sand and 30% tire chips by weight, and the authors reported that the use of shredded tires/Ottawa sand as a lightweight filler material has a promising effect. Furthermore, the authors suggested that the maximum size of tire chips used should be 4.75 mm^54–56^. An analytical investigation was conducted to evaluate the performance of the raft foundation with the addition of piles in it using PLAXIS 2D software package. From the investigation, the authors reported that an increase in the number of piles reduces the settlement of the footing. Furthermore, the researchers reported that a constant range in the reduction in a settlement is observed with a further increase in plies. The new method for seismic isolation, called a distributed isolation system, by placing the rubber soil mixture below the footing, was analytically studied for ten-storey buildings. The authors concluded that the proposed method effectively reduces the horizontal and vertical accelerations by 60–70% and 80–90%, respectively^57–59^. The experimental investigation was conducted to test the behaviour of a model footing resting on a sand-rubber layer. The entire setup was placed in a plexi glass block, mounted on a shake table and subjected to sinusoidal motion. The authors reported that the proportion of RSM with 50% rubber by weight is the effective base isolation combination^60^. The finite element analysis was conducted to investigate the seismic response of soil–tire mix in various ratios such as 0%, 10%, 20%, 30% and 50% using ABAQUS software, and the soil system is subjected to earthquake excitations (Time History Analysis). The authors concluded that a reduction in the acceleration of about 40% is attained with the thickness of the isolating layer as two times the width of the footing^61^.

The research is categorized into two phases. The performance of the footing and the building subjected to cyclic loading respectively is evaluated. The soil–tire mix with 30% tire crumbs is applied for the study and compared with that of 0% tire crumbs. To be concise, the performance of the footing with and without tire crumb base isolation is broadly studied. In phase 1, cyclic loading is applied on a model footing placed on sand (with and without tire crumbs) and subjected to cyclic loading using a plate load test. In the second phase of the research, seismic loading is applied on a four-story residential building situated on the Nepal border of India^62–64^. The acceleration of the building is determined for the selected seismic excitations for the study.

## Materials and methods

### Experimental evaluation using plate load test

The soil taken from the regions of Avadi-Chennai has been employed in this study. The type of soil was confirmed using a sieve analysis. Based on the results of this initial study, it is determined that the soil is a poorly graded according to the Indian geotechnical code, Indian Standard (IS): 1498–1970^57^. The scrap tires were well processed, removing the steel imbedded over them and free from dirt to tire crumbs from a well-advanced scrap tire processing plant situated in Ambattur Estate, Chennai. Before the commencement of the study, it is ensured that the tire crumb (TC) passes through 4.75 mm IS sieve^56–61^. The geotechnical properties of the clayey soil and soil–tire mix in the case of 0% TC and 30% TC are shown in Table 1.

**Table 1:** The geotechnical properties of the clayey soil and soil–tire mix in the case of 0% TC and 30% TC.

Parameters	Types of soil		
	Clayey soil	soil–tire mix in the case of 0% TC	soil–tire mix in the case of 30% TC
Unsaturated unit weight	18 kN/m^3^	16 kN/m^3^	11 kN/m^3^
Saturated unit weight	22 kN/m^3^	19 kN/m^3^	15.8 kN/m^3^
Young’s modulus	20 (MPa)	20 (MPa)	20 (MPa)
Poisson ratio	0.2	0.2	0.2
Cohesion	10 kN/m^2^	20 kN/m^2^	4 kN/m^2^
Friction angle	18°	23.72°	28.50°
Depth of the layer	0.4 m	25 mm	0.4 m

**Figure 2:**
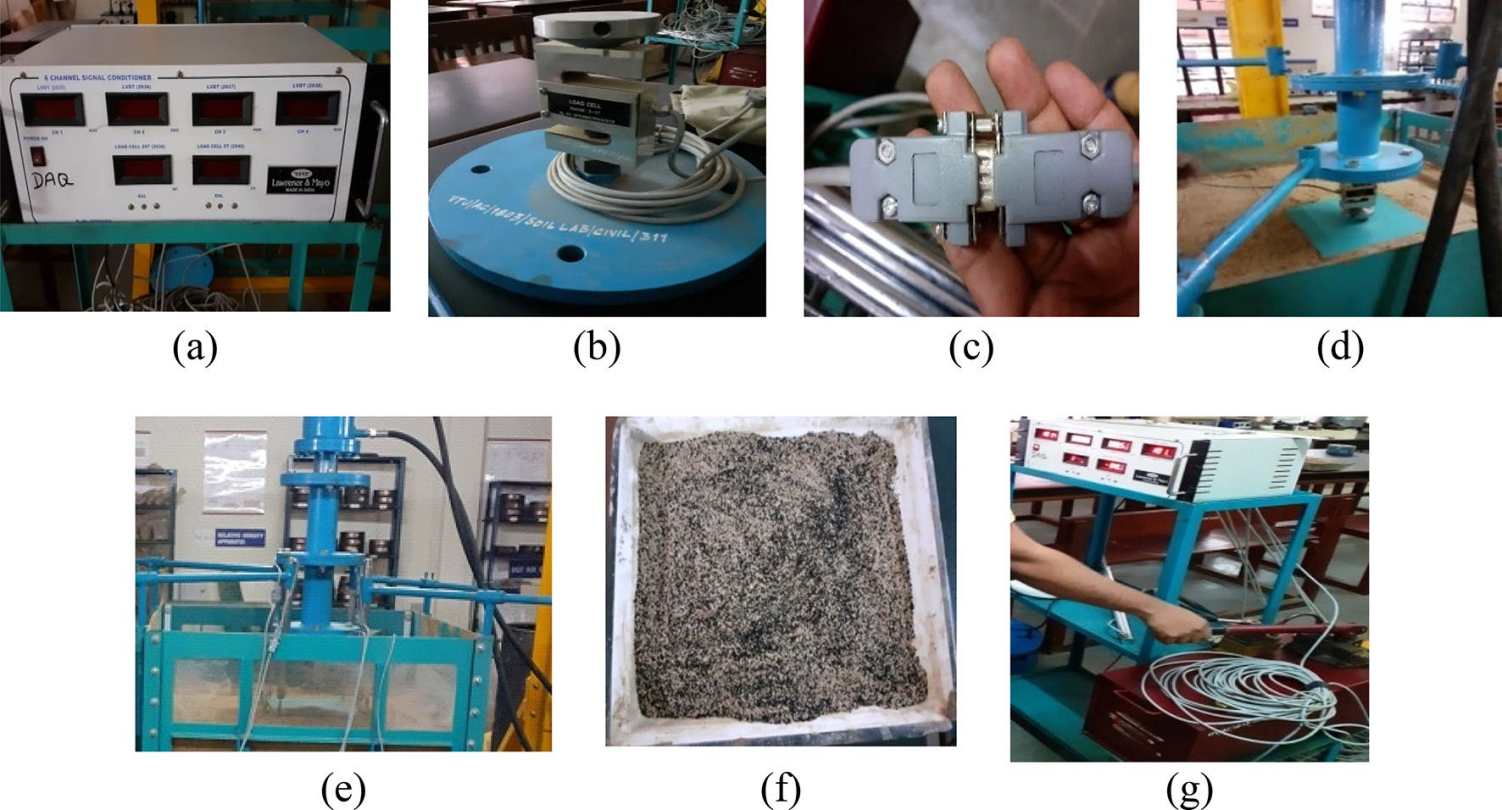
Experimental setup (**a**) Reading box (**b**) 5 ton load cell (**c**) LVDT connection (**d**) Tank with footing (**e**) Test tank (**f**) soil–tire mix (**g**) Application of load.

The researchers have proposed that the 30% combination of a tire with soil furnishes satisfactory damping properties and shear strength irrespective of higher percentages of tire crumbs^50^. Therefore, for this research, the efficiency pertaining to 30% tire crumb combination has been broadly investigated. Though the soil comes under the category of poorly graded, the influence of tire crumbs ultimately equalizes the shear strength criteria. Moreover, the researchers reported that the possibility of liquefaction is prevented with the inclusion of tire crumbs with the soil^51^. Triaxial compression tests have been conducted for the soil, and soil–tire mix combinations maintained with a relative density of 85%.The soil–tire mix is placed in a mold for five layers and thoroughly tamped. The researchers reported that segregation in the soil–tire mix is relatively low^48^. The pictorial representation of the experimental setup is shown in Fig. 2.

The entire setup consists of a metal test tank with four dial gauges connected to the respective LVDT’s. In addition, a mechanically operated pump is fixed to the setup for load application. Based on the shear strength factor of the soil and footing rigidity, the size of the model footing is taken^51^. Moreover, based on the availability of the footing sample, a model footing of size 0.3 m × 0.3 m × 0.05 m is used for the investigation. The metal tank is thoroughly cleaned to free it from dirt and other particles. The experiment is carried over in two different phases, as shown in Fig. 2a,b. Based on the concept of pressure bulb (0.5 B), B is the width of the footing, and the metal tank is filled with two different types of soil^55^. In both phases, the metal tank is for instance, filled with soils in layers with each layer to be 0.1 m and eventually, up to 0.8 m, the tank is filled. Clayey soil is placed upto the depth of 0.4 m and subsequently, sand which is of poorly graded soil is filled from 0.4 to 0.8 m. It is to be noted that the perfect compaction of the layers is accomplished to attain a relative density of 85%. A 5-to-load cell is placed for the application of load.

The entire experiment is conducted using a plate load test setup. In the first part, an experiment is conducted without soil–tire mix. The performance pertaining to 0% tire crumb is evaluated as shown in Fig. 2a. Subsequently, the experiment is performed with a soil–tire mix as shown in Fig. 2b. The performance pertaining to 30% tire crumb as recommended by the researchers^56,57^ is evaluated. soil–tire mix is placed to a depth of 25 cm^48^. Load is applied and correspondingly applied for the reverse cycles. Load versus deflection graphs are plotted, and the respective deformation is noted. From the load versus deflection graphs, the equivalent stiffness and energy dissipation capacity of the footing is determined (Fig. 2a–g).

### Investigation using PLAXIS 2D software

**Figure 3:**
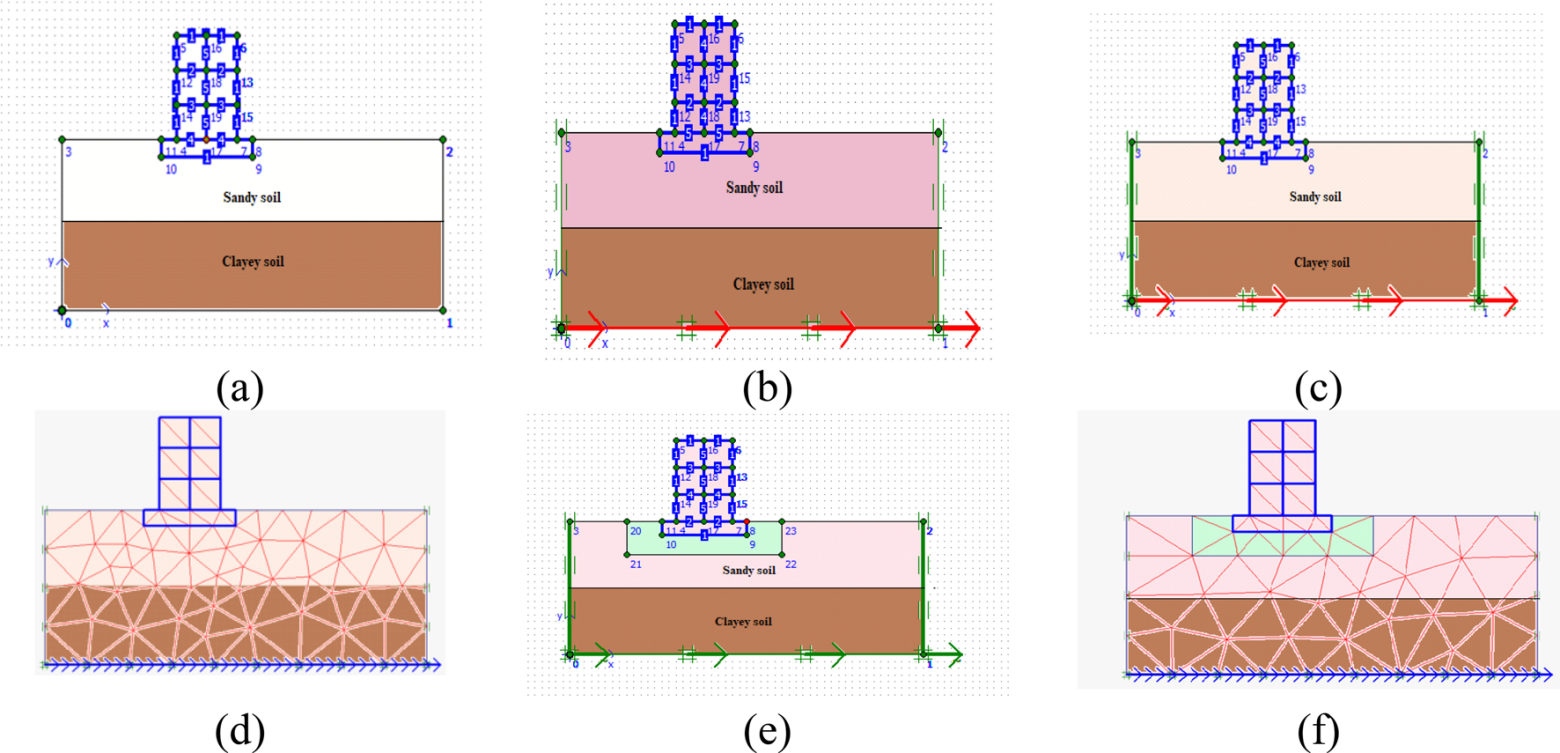
Finite Element analysis (**a**) Modelling (**b**) Assigning prescribed displacement (**c**) Applying of earthquake boundaries (**d**) Meshing with subsoil layer (**e**) Placing of soil–tire layer (**f**) Meshing with soil–tire layer.

The investigation consists of dynamic analysis of a three- storied building placed on the layers of sandy soil, subsoil (Clayey soil) in first case and additionally soil–tire mix in the second case. For instance, the geotechnical tests such as triaxial compression test, specific gravity test and sieve analysis is conducted. From the sieve analysis test, it is observed that the type of soil in the top layer for the study is a poorly-graded soil. The analysis is performed for the building without and with base isolation (soil–tire mix) for 70% soil and 30% tire crumbs^47^. The depth of tire soil mix considered for the analysis is 10 m. The two cases (with and without base isolation) are subjected to earthquake excitations with a very prominent disastrous events such as Northridge earthquake (1994), Landers earthquake (1992) and Upland earthquake (1990) (Peer Ground Motion Database and COSMOS Ground Motion Database)^51^. A brief procedure of finite element modelling, meshing and earthquake applications are shown in Fig. 3a–f**.**

In order to prevent mesh locking effects for almost incompressible materials, the elements utilized are adequately sufficient in the investigation. This unique feature has been applied for the modelling of undrained material behaviour based on effective model parameters for the majority of material models. Therefore this makes it possible to do undrained calculations with effective stiffness parameters and clearly distinguished the effective stresses from (excess) pore pressures. Moreover, 15 noded triangular elements are applied in the study. Mohr–Coulomb model has been implemented in the investigation. In actuality, the stress level, the stress path, and the strain level are the minimum factors that determine how stiff the soil is. The PLAXIS advanced soil models contain some of these features. However, the well-known and straightforward Mohr–Coulomb model is a linear elastic completely plastic model has been applied. Hooke's law of isotropic elasticity serves as the foundation for the linear elastic portion of the Mohr–Coulomb model. Based on the Mohr–Coulomb failure criterion, which was developed in a non-associated plasticity framework, is the perfectly plastic portion. For instance, in the PLAXIS 2D finite element analysis, the geometry lines are drawn to complete the subsoil layer, tire soil mix and the building. The building is a three-storied building with each floor to be 3 m high. The width of the building is 3 m^50–53^. The depth of the footing considered is 2 m. The building and the footing are the plate elements and the analysis is linear elastic since it is of dynamic earthquake analysis (COSMOS Ground Motion Database)^63^. The material properties of the soil, tire-soil mix and the plate elements were fed in the software and mesh generation is accomplished. The calculation consists of two phases such as plastic and dynamic phases. In plastic phase, all the plate elements are activated and the soil clusters are deactivated. In dynamic phase, the corresponding earthquake time history plots are the applied (COSMOS Ground Motion Database)^50^.

## Results and discussion

### Load versus deflection plots

**Figure 4:**
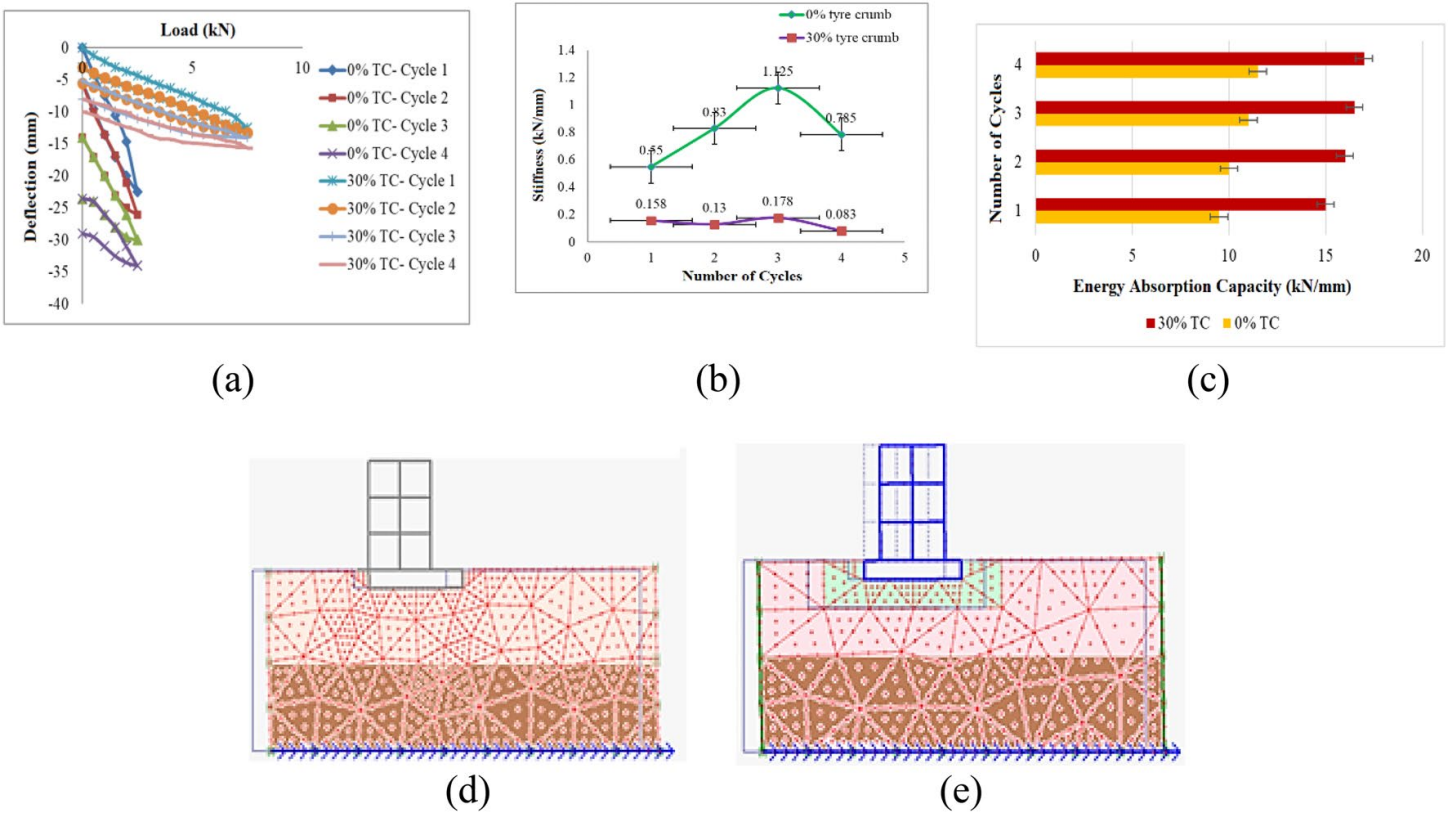
Performance of the Frame (**a**) Load versus deflection plot (**b**) Reduction in stiffness (**c**) Increase in energy absorption capacity (**d**) Deformed mesh without TC (**e**) Deformed mesh with TC.

The loading is applied in cyclic pattern into four different cycles under incremental loads. Hand-operated pump is applied for loading and the load value is displayed on load-reading box. Through the LVDT’s placed at the corners of the footing, the corresponding deflections are noted in each cycle. At the end of the fourth cycle, the entire footing fails. For 30% TC, the footing fails at 7.5 kN load and for 0% TC, the footing fails at 2.5 kN which is the significant aspect to be observed. Similar range of results are observed in the literatures^17^. The respective load–deflection graph is plotted as shown in Fig. 6. From the graph, the energy absorption capacity and the degradation in the stiffness of the footing is assessed. The respective load–deflection graph is plotted as shown in Fig. 4a. From the graph, the energy absorption capacity and the degradation in the stiffness of the footing is assessed.

For 0% tire crumb with soil, the development of settlement is quite earlier compared to 30% tire crumb combination as reported by the researchers^42^. The damping property of the tire crumbs significantly delay the failure of the footing. The degradation in stiffness ultimately increases the fundamental period of the building which is the required aspect in seismic engineering and represented in From Fig. 4b for this investigation.

At the end of the fourth cycle, the stiffness degradation is 0.785 kN/mm in case of 0% TC and 0.083 kN/mm in case of 30% TC. From this, it is evident that tire crumbs mixed with soil with 30% have significant reduction in frequency and increase in period^53–58^. The stiffness reduction for 30% TC for the first cycle is 0.158 kN/mm, which is 75.69% higher than the stiffness degradation value for 0% TC obtained from the study, which is 0.65 kN/mm. The stiffness reduction for 30% TC for the second cycle is 0.13 kN/mm, which is 84.33% more than the stiffness value obtained from the study of 0.83 kN/mm for 0% TC. The reduction in stiffness for 30% TC for third cycle is 1.125 kN/mm which is 84.17% greater than 0%TC with its stiffness value 0.178 kN/mm attained from the study. The stiffness reduction for 30% TC for the third cycle is 0.785 kN/mm, which is 88.12% more than the stiffness value obtained from the study for 0% TC, which is 0.083 kN/mm and eventually the stiffness increases for further loading and the footing fails. From the results it is observed that stiffness degradation is prominent 30% TC compared to 0% TC due to the damping property of TC during 30% TC combination and similar pattern of results are reported in the literatures^59^.

### Energy absorption capacity

The substantial role of the seismic base isolation material is the capacity to absorb the earthquake energy so that the superstructure is protected. Figure 4c shows the increase in energy absorption capacity and number of load cycle sand types of soil with TC 0% and TC 30%. The area enclosed in the loop is eventually evaluated for the determination of the energy dissipation capacity. From the cyclic loading plot for each cycle and for both the tire crumb (TC) combination (0% TC and 30% TC), the energy dissipation capacity is determined. For the first cycle, the percentage increase in energy absorption capacity for 30% TC is 57.89% greater than the value for 0% TC. In case of second cycle's energy absorption capacity for 30% TC is 70.12% greater than 0% TC. The increase in energy absorption capacity for 30% TC during the third cycle is 72.73% more than 0% TC.

Subsequently, for the fourth cycle, the percentage increase in energy absorption capacity is 50.12% for 30% TC compared to 0% TC and eventually the absorption capacity drops due to the failure of the footing. Due to the damping feature of TC when 30% TC is combined, the results show that energy absorption capacity is more pronounced at 30% TC than at 0% TC, and similar results are documented in the literature^59,60^. Therefore, 30% TC have satisfactory energy dissipation capacity compared to 0% TC. Similar pattern of results is reported by the researchers^54–56^.

### Acceleration and deformed mesh

The pattern of deformed mesh with 0%TC, the building failures significantly noted at the footing. In contrast, with 30% TC, the sliding of the building is observed as the tire crumbs act as a cushion and enhance the sliding of the building preventing transfer of seismic waves into the superstructure. The cumulative energy dissipation at the end of the fourth cycle is determined as 42 kN/mm and 64.5 kN/mm respectively for 0% TC and 0% TC respectively. For instance, with 0% tyre crumb isolation system, the peak acceleration is found to be 0.45 m/s^2^, 0.8 m/s^2^ and 0.224 m/s^2^ for Northridge earthquake (1994), Landers earthquake (1992) and Upland Earthquake (1990) respectively. With tyre crumb isolation system, the reduction in acceleration of about 0.25 m/s^2^, 0.62 m/s^2^ and 0.141 m/s^2^ respectively. The magnitude, PGA, and frequency of the components have a significant impact on how quickly the building accelerates. The literatures^64–68^ show a similar pattern of results: the greater the magnitude, the less the acceleration is reduced. Compared to other earthquakes, the SE 2 (Northridge earthquake) event exhibits a more pronounced drop in acceleration. Due to low PGA in the earthquake, which lowers the frequency of the buildings, SE 3 (Upland earthquake 1990) has fewer declines in acceleration than other occurrences. The literature^68^ reveals a similar pattern of outcomes. Similar outcomes are observed and documented in the literature^62–70^. The use of tyre crumbs to boost damping property reduces the rigidity of the structure, which in turn lowers frequency. The deformed mesh with and without TC is shown in Fig. 4e,f, respectively.

**Figure 5:**
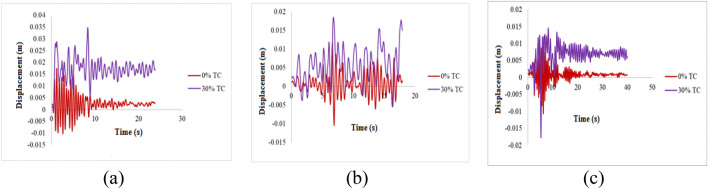
Displacement Plot (**a**) Northridge earthquake (1994) (**b**) Landers earthquake (1992) (**c**) Upland Earthquake (1990).

**Figure 6:**
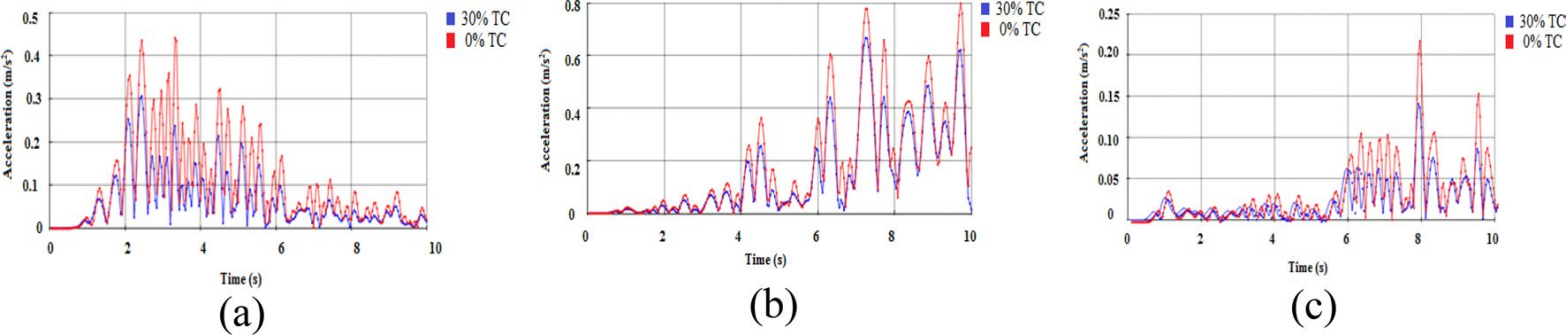
Acceleration Plot (**a**) Northridge earthquake (1994) (**b**) Landers earthquake (1992) (**c**) Upland Earthquake (1990).

The buildings' flexibility is significantly increased by the added story displacement brought about by the installation of isolators, and the isolator can slide. Compared to occurrences SE 2 and SE 1, the displacement of SE 3 is significantly higher. Due to the use of tyre crumbs as the base isolation material, the PGA of the events significantly contributes to the building's increased displacement, and comparable patterns of outcomes are seen in the literature^70^. The displacement plot with respective to three different earthquakes are shown in Fig. 5a–c. Similarly, the acceleration plot with respective to three different earthquakes are shown in Fig. 6a–c.

**Figure 7:**
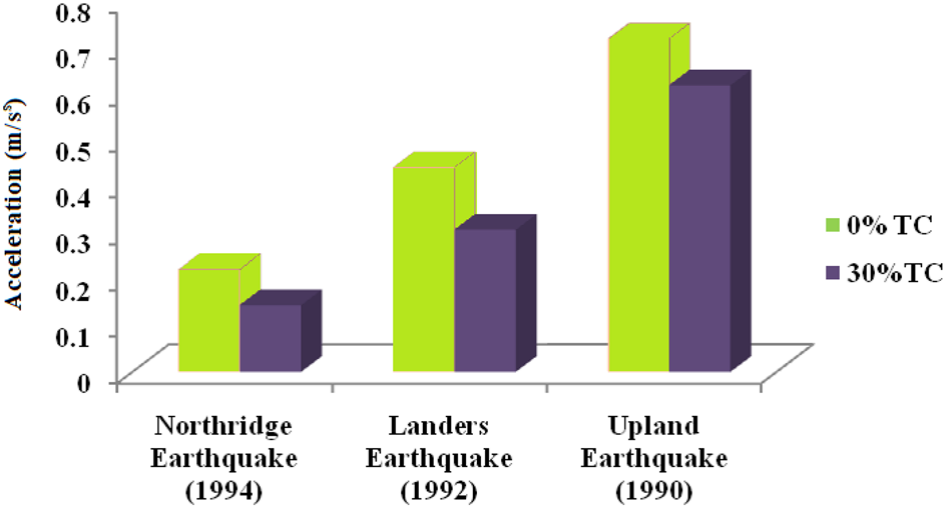
Reduction in acceleration.

From the evaluation, the acceleration significantly reduces from 0.221 to 0.144 m/s^2^ for Northridge Earthquake (1994), 0.441–0.306 m/s^2^ for Landers Earthquake (1992) and 0.719–0.618 m/s^2^ for Upland Earthquake (1990) with TC mix in soil (Fig. 7).

## Conclusion

The implementation of seismic isolation in an eco-friendly way could be significantly adopted by the usage of recyclable tire crumbs. The modern world seeks to mitigate the effect of seismic events irrespective of cost. Nevertheless, for a developing country the conventional method of seismic base isolation is yet to be fulfilled. Despite of cost, safety is a key factor to be considered for seismic protection. The adoption of this tire-soil method of base isolation has the capacity in reducing the acceleration of the structures (buildings) with about 30% to 40% effectively. Therefore, the main scope of this paper to bring out the idea of the usage of hazardous tire wastes/industrial by-product in the field of civil engineering (base isolation technology) is vital as each and every life is important and it is the sole duty of each and every individual to protect our ecosystem. From the investigation, following conclusions are drawn (Supplementary information).

- The increase in tire crumbs with 30% TC, it is observed that the footing fails under greater loads.
- The degradation of stiffness with about 1.7–1.8 10times greater is attained with the inclusion of tire crumbs with soil (30%TC) compared to 0% TC.
- Significant energy absorption capacity of about 50–90% is observed with 30%TC mix in the soil compared to 0% TC.
- An acceleration reduction of about 1.6 times to 1.7 times be achieved with 30% TC compared to 0% TC during SE 3 compared to SE 2 and SE 1 respectively.
- An exclusive increase in displacement of about 42–50% is observed in SE 3 compared to SE 2 and SE 1 respectively.
- Hence, it is concluded that tire crumbs mixed with soil with 30% is exclusively suitable for base isolation of low to medium rise buildings.
- The main limitation of this isolation system method is that it requires substantial study before being applied to live buildings or structures. Expected geotechnical issues include liquefaction, shear strength, location of the water table, soil characteristics, and bearing capacity. The price of the tyre crumbs is reasonable, and they are readily available. However, estimating the total amount of tyre crumbs for all real-time structures is a difficult process that necessitates much investigation. The tyre scraps should be buried beneath the foundation. Therefore, even using jacking techniques to replace the crumbs for repair in the future would be laborious because it would damage the foundation of older structures.

### Supplementary Information

Supplementary Information.

## Data Availability

The datasets used and analyzed during the current study are available from the corresponding author on request.
