# Supplementary material for: Numerical modelling on geotechnical features of soil mixture using recycled tire crumb to strengthen the seismic isolation in building
Source: Sci Rep. 2024 Jan 2;14:225. doi: 10.1038/s41598-023-50741-w (PMC10762241; doi:10.1038/s41598-023-50741-w)
Supplement: Supplementary file 1 — Supplementary Information. [file 41598_2023_50741_MOESM1_ESM.docx]

**Supplementary Table S1.** Properties of the building for finite element analysis

| Parameters Considered | Corresponding values |
| --- | --- |
| Normal Stiffness | 5E6 kN/m |
| Flexural Rigidity | 9000 kN/m^2^/m |
| Rayleigh dampers (α and β) | 0.01 |
| Weight | 5 kN/m/m |

**Supplementary Table S2.** Seismic Events ^50^

| Seismic Event | Seismic Event | Year of the Event | Station | Magnitude | PGA (g) | Hypocentral distance (km) |
| --- | --- | --- | --- | --- | --- | --- |
| SE1 | Northridge Earthquake | 1994 | San Gabriel, CA | 6.8 | 1.82 | 38.8 km |
| SE2 | Landers Earthquake | 1992 | Barstow, CA | 7.5 | 0.16 | 44.4 km |
| SE3 | Upland Earthquake | 1990 | Pomona | 5.2 | 1.05 | 11.7 km |


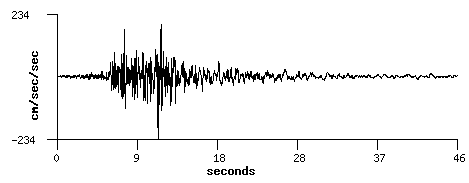


(a)


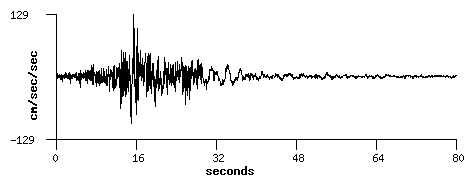


(b)


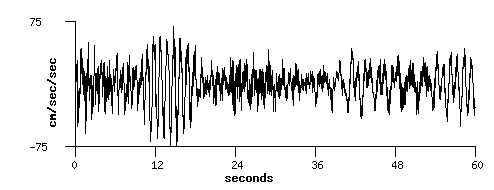


(c)

**Supplementary Figure. S1** Acceleration Time History (a) Northridge earthquake (1994) (b) Acceleration time history of Landers earthquake (1992) © Acceleration time history of Upland Earthquake (1990)^50^
